# Type I IFNs Decrease SARS-CoV-2 Replication in Human Cardiomyocytes and Increase Cytokine Production in Macrophages

**DOI:** 10.1007/s10875-025-01943-6

**Published:** 2025-10-21

**Authors:** Verónica Durán, Eirini Nikolouli, Shambhabi Chatterjee, Bibiana Costa, Andreas Pavlou, Annett Ziegler, Jennifer Becker, Kira Baumann, Matthias Bruhn, Kathrin Haake, Anna Rafiei Hashtchin, Ingrid Gensch, Andrea Korte, Yvonne Lisa Behrens, Shen-Ying Zhang, Jean-Laurent Casanova, Christian Bär, Nico Lachmann, Thomas Thum, Ulrich Kalinke

**Affiliations:** 1 Institute for Experimental Infection Research, TWINCORE, Centre for Experimental and Clinical Infection Research, a joint venture between the Helmholtz Centre for Infection Research [HZI], Braunschweig, and the Hannover Medical School [MHH], 30625 Hannover, Germany; 2 Department of Microbiology and Immunology, Stanford University School of Medicine, Stanford, 94305 CA USA; 3 Chan Zuckerberg Biohub, CA San Francisco, USA; 4 Department for Pediatric Pneumology, Allergology and Neonatology, Hannover Medical School [MHH], 30625 Hannover, Germany; 5 Institute of Molecular and Translational Therapeutic Strategies, Hannover Medical School [MHH], 30625 Hannover, Germany; 6 Center for Translational and Regenerative Medicine, Hannover Medical School [MHH], 30625 Hannover, Germany; 7 Institute of Experimental Hematology, Hannover Medical School [MHH], 30625 Hannover, Germany; 8 Department of Human Genetics, Hannover Medical School [MHH], 30625 Hannover, Germany; 9 Laboratory of Human Genetics of Infectious Diseases, Necker Branch, INSERM U1163, Paris, France; 10 Imagine Institute, Paris University, Paris, France; 11 St. Giles Laboratory of Human Genetics of Infectious Diseases, Rockefeller Branch, Rockfeller University, New York, USA; 12 Pediatric Immunology-Hematology Unit, Assistance Publique-Hospitaux de Paris [AP-HP], Necker Hospital for Sick Children, Paris, France; 13 Howard Hughes Medical Institute, New York, NY USA; 14 Fraunhofer Institute for Toxicology and Experimental Medicine [ITEM], 30625 Hannover, Germany; 15 Cluster of Excellence - Resolving Infection Susceptibility [RESIST, Hannover Medical School, EXC 2155], Carl-Neuberg-Straße 1, 30625 Hannover, Germany

## Abstract

The cellular basis of COVID-19 severity in patients with deficiencies in type I IFN immunity remains unclear. In this study, we differentiated cardiomyocytes and macrophages from IFNAR1 competent (IFNAR1^comp^) and deficient (IFNAR1^def^) induced pluripotent stem cells (iPSCs), and analyzed virus replication and cytokine production after exposure to SARS-CoV-2. Cardiomyocytes expressed the SARS-CoV-2 receptor angiotensin-converting enzyme 2 (ACE2) and showed abundant SARS-CoV-2 replication, which was higher in IFNAR1^def^ than IFNAR1^comp^ cells. Treatment with exogenous IFNα mitigated infection in IFNAR1^comp^, but not in IFNAR1^def^ cardiomyocytes. In contrast, macrophages did not express ACE2 and did not support SARS-CoV-2 replication, but produced pro-inflammatory cytokines upon virus exposure, which was impaired in IFNAR1^def^ macrophages. In conclusion, type I IFNs decrease SARS-CoV-2 replication in human iPSC-derived cardiomyocytes, while they increase cytokine responses of macrophages.

**Supplementary Information:**

The online version contains supplementary material available at 10.1007/s10875-025-01943-6.

## Introduction

The COVID-19 pandemic, declared on March 11, 2020 [1], has dramatically reshaped our understanding of viral diseases and immune responses. Despite significant advances, the intricate mechanisms driving the varied clinical outcomes of infection with severe acute respiratory syndrome coronavirus-2 (SARS-CoV-2) to a large extent remain unclear. While most individuals experience mild symptoms, approximately 20% develop pneumonia [2, 3]. Half of the pneumonia cases are moderate, i.e., they are non-hypoxemic, whereas 10% of the patients face critical hypoxemic pneumonia, with a global fatality rate of approximately 1% [4]. In some patients, pneumonia is accompanied by dysregulated cytokine production and increased inflammation, which can result in complications such as myocarditis [5] and multisystem inflammatory syndrome in children (MIS-C) [6]. Thus, understanding determinants of the high variability in COVID-19 symptoms and disease severity is crucial for the development of effective prevention and treatment strategies.

Risk factors for life-threatening COVID-19 include male sex, pre-existing medical conditions and age, which is the major epidemiological determinant for hospitalization and death [7–11]. These risk factors often correlate with impaired type I interferon (IFN-I) responses [12–14]. In men under 60 with severe COVID-19 pneumonia, approximately 1% have impaired IFN-I production due to X-linked deleterious mutations in TLR7, an immune sensor involved in the induction of IFN-I [15]. Autoantibodies neutralizing IFN-I are present in approximately 4% of uninfected individuals over 70 years old, and account for approximately 20% of COVID-19 deaths [16–18]. Interestingly, pulmonary SARS-CoV-2 viral loads appear to be similar in patients with or without these autoantibodies [19], suggesting that IFN-I autoantibodies may not substantially impact early viral control, but may contribute to disease by altering the downstream immunomodulatory functions of IFN-I.1–5% of patients with life-threatening COVID-19 pneumonia had inborn errors in the IFN-I axis, including TLR3- or TLR7- and IRF7-dependent induction of IFN-I [12, 20, 21]. Serum levels of soluble IFNAR1 were inversely correlated with COVID-19 severity [22]. Importantly, autosomal recessive deficiency of the IFN-I receptor 1 chain (IFNAR1) was detected in unrelated critical cases of COVID-19 pneumonia [20, 23], and in vitro studies with IFNAR1 deficient fibroblasts revealed their increased susceptibility to SARS-CoV-2 infection [24].

SARS-CoV-2 infection can result in a multiorgan disease, predominantly affecting the lungs, and characterized by fibrosis, alveolar damage, extensive inflammation, and increased macrophage infiltration [25–29]. Macrophages and plasmacytoid dendritic cells (pDC) are not productively infected by SARS-CoV-2 [30, 31], but act as potent cytokine producers in response to infection [32–35], thus contributing to the hyperinflammatory phenotype in COVID-19 patients. While the function of macrophages during onset and progression of COVID-19 is yet to be fully elucidated and may change depending on the context of disease, the impact of infection on cardiovascular cell types is better defined. Cardiomyocytes expressing high levels of angiotensin-converting enzyme 2 (ACE2) [36, 37] — a key SARS-CoV-2 entry receptor [38] — are highly susceptible to infection [39–41]. This correlates with increased markers for cardiac damage and dysfunction, as well as myocarditis in severely sick COVID-19 patients [42–45].

Overall, understanding the cellular basis by which defects in the IFN-I axis cause critical disease is of clinical and biological relevance. These defects may impact defined cell subsets in different ways, either by directly altering SARS-CoV-2 infection and replication, or by indirectly affecting inflammatory responses and disease exacerbation. Our study aimed at unraveling the role of IFN-I immunity during SARS-CoV-2 infection in human macrophages (MACs) and cardiomyocytes (CMs). By generating hiPSC-MACs and hiPSC-CMs from IFNAR1 competent (IFNAR1^comp^) and IFNAR1 deficient (IFNAR1^def^) individuals, we demonstrated how IFN-I responses disturb SARS-CoV-2 replication and promote cytokine production, thus influencing the development of critical COVID-19 pneumonia.

## Materials and Methods

### Ethical Statement

Human fibroblasts were collected from a patient with IFNAR1 deficiency in Israel [46]. Informed consent was in accordance with local regulations and the requirements for institutional review board approval from The Rockefeller University and Institut National de la Santé et de la Recherche Médicale (INSERM).

### Reprogramming and Cultivation of IFNAR1 ^def^ hiPSCs

IFNAR1^def^ human fibroblasts harboring the mutation Chr21(GRCh37):g.34,726,420_34,728,094del [46] were subjected for reprogramming as described elsewhere [47]. For this, a “four-in-one” 3rd generation SIN lentiviral vector containing the original Yamanaka factors was used [48]. Three to five days after the viral transduction, the cells were transferred and cultured on irradiated murine CF1 feeder cells. Upon colony formation, several ones were picked and cultured for the establishment of clones in human iPSC-medium [knock-out Dulbecco’s modified Eagle medium supplemented with 20% knock-out serum replacement, 1% penicillin/streptomycin, 0.1 mM beta-mercaptoethanol, 1 mM L-glutamine, 1% nonessential amino acids and 20 ng/ml bFGF (Invitrogen)]. While IFNAR^comp^ hiPSCs were generated using a non-integrating Sendai virus approach, previous studies have highlighted the comparable quality and functionality of hiPSC-derived cells, regardless of the reprogramming method used [49, 50]. Importantly, the IFNAR^def^ hiPSCs were intentionally derived from a patient with documented increased susceptibility to viruses who was found to have a distinctive form of inherited IFNAR1 deficiency [46], providing a clinically relevant context for our study.

### Chromosomal and Short tandem Repeat Analysis of IFNAR1 ^def^ hiPSCs

Chromosome preparation and fluorescence R-banding were performed as previously described [51]. Whenever possible, 15 metaphases per iPSC clone were analyzed. The karyotype was described according to the guidelines of the International System for Human Cytogenetic Nomenclature (Fig. S1a).

To confirm the identity of the IFNAR^def^ hiPSC line and the absence of contamination with any other human DNA or hiPSCs, genetic profiling was performed using highly polymorphic short tandem repeat (STR) loci. STR loci were amplified using the PowerPlex^®^ 16 HS System (Promega) and fragment analysis was done on an ABI3730xl (life Technologies). The obtained data were analyzed with GeneMarker HID software (Softgenetics). The analysis confirmed the genetic identity of the IFNAR^def^ hiPSC line with the original patient-derived fibroblasts and verified the absence of any contamination (Fig. S1 B).

### Generation of hiPSC-CMs

IFNAR1^comp^ (hHSC_Iso4_ADCF_SeV-iPS2, alternative name: MHHi001-A) [52] and IFNAR1^def^ hiPSC lines were maintained under feeder-free culture conditions. For generation of hiPSC-derived CMs we used a well-established protocol by modulating the Wnt pathway [53]. Briefly, hiPSCs were cultured with Cardio Differentiation media [RPMI GlutaMAX medium (Gibco), albumin human recombinant (Sigma-Aldrich) and L-Ascorbic acid 2-phosphate sesquimagnesium salt hydrate (Sigma-Aldrich) supplemented with 5 µM of GSK3β inhibitor CHIR99021] to reach a cellular density of 70–80%. At day 2, medium was exchanged with fresh medium supplemented with 5 mM of the Wnt signaling inhibitor IWP2 (Peprotech). Medium was exchanged at day 4 and 6, and from day 8 on, cells were cultured in RPMI GlutaMAX medium supplemented with B27 (Gibco), with medium changes every 2 days. Between day 15–20, hiPSC-CMs were enriched by metabolic purification with lactate (Sodium-DL-lactate, Sigma), and maintained in culture for maturation until day 60 using RPMI GlutaMAX medium supplemented with B27 as described previously [54].

### Generation of hiPSC-MACs

IFNAR1^comp^ (cl16: hCD34iPSC16) [55] and IFNAR1^def^ hiPSC lines were expanded on irradiated murine CF1 feeder cells. Upon embryoid body formation, these were picked and transferred into an adherent 6-well plate with X-Vivo differentiation medium (Lonza) enriched with 1% penicillin/streptomycin, 1 mM L-glutamine, 0.05 mM beta-mercaptoethanol, 50 ng/ml M-CSF (Peprotech) and 25 ng/ml IL-3 (Peprotech), and left to attach. Medium exchange was performed once per week. After 2 weeks in culture, the first monocytic cells were harvested and cultured for final differentiation for 3–5 days in RPMI medium supplemented with 10% fetal calf serum (FCS) (Sigma), 1% penicillin/streptomycin and 50 ng/ml M-CSF [56, 57].

### Flow Cytometry

For ACE2 labeling, 0.2 × 10^6^ CMs were harvested by washing with Accutase (StemcELL) and MACs with 1x PBS, followed by filtering through 100 μm strainers. Both cell types were resuspended in labeling buffer [PBS containing 2 mM EDTA and 10% BSA] with Fc-block (Gamunex 10%, Grifols) and incubated for 10 min at RT. Anti-human ACE2 (AC384; Novus Biologicals) or the respective isotype control antibody (P3.6.2.8.1; Novus Biologicals) were used as primary antibodies, together with a secondary anti-mouse antibody conjugated with AlexaFluor 647 (A-31571, ThermoFisher Scientific). For live/dead discrimination, propidium iodide (Biolegend) was added at dilution 1:20 for 15 min at RT in the dark. Antibody labelings were performed for 20 min at 4 °C in the dark, and cells were washed with FACS buffer [PBS containing 20 mM EDTA, 2% BSA and 0.2% sodium azide]. Cell acquisition was performed on a SONY ID7000 spectral flow cytometer. For cell surface profiling of MACs, the following antibodies were used: CD11b (25-0118-41, eBioscience), CD14 (11-0149-41, eBioscience), CD45 (17-0459-42, eBioscience), CD163 (12-1639-41, eBioscience), HLA-DR (307609, BioLegend). Cell acquisition was performed on a CANTO II (BD Biosciences) spectral flow cytometer. Data were analyzed by using FlowJo software (TreeStar).

### Phagocytosis Assay

The phagocytic capacity of MACs was addressed using the pHrodo Red *S. aureus* BioParticles Conjugate (MolecularProbes/Thermo Fisher Scientific). Incubation at 4 °C in medium without BioParticles were used as negative controls. Upon certain time points, the cells were collected with cold 1x PBS and analyzed by flow cytometry. Cell acquisition was performed on a CANTO II (BD Biosciences) spectral flow cytometer. Data were analyzed using FlowJo software (TreeStar).

### RNA and Real-time PCR

RNA was isolated using TriFast (Peqlab) and its concentration was measured using Take3 Plates on a Bio-Tek plate reader (Synergy HT). For mRNA measurements, RNA (1,000 ng) was reverse transcribed using the Biozym cDNA synthesis kit. Real-time PCR was performed with iQ SYBR Green mix [Bio-Rad] in a ViiA7 (Applied Biosystems) machine using specific primer pairs for the CMs and MACs (see table below).

**Table:** 

Target	Forward primer sequence (5’->3’)	Reverse primer sequence (5’->3’)
*h_ACE2*	GGACCCAGGAAATGTTCAGA	GGCTGCAGAAAGTGACATGA
*h_TBP*	CCACTCACAGACTCTCACAAC	CTGCGGTACAATCCCAGAACT
*h_NRP1*	GCCACAGTGGAACAGGTGAT	ATGACCGTGGGCTTTTCTGT
*h_TNNI1*	GGGTGACTGGAGGAAGAACG	CAGGCTGGAGGGAAGAAGTG
*h_RYR2*	AAAGAGCTCAGTGTGGACGA	AGGCTTGCATGACTTCGTTG
*h_MYH7*	CCTGCTCTGTGTCTTTCCCT	ACTGCCATCTCCGAATCTCC
*h_MYH6*	GTGGACAAGCTGCAACTGAA	GTCACTCCTCATCGTGCATT
*h_IRX4*	TCTACTGCCCGGTCTACGAG	GCAGATCCCGAACCATCCTTG
*h_HCN1*	GGGGAAAGCAGTATTCATACGC	ATGGTAATCCAGAGGTCAGACA
*h_CORIN*	CCTCCTCCGGTTCCTATTGC	CCAAAGGTTCACTCCCATTTGA

### Viruses

The SARS-CoV-2 isolate used in this study (BetaCoV/Germany/BavPat1/2020; EPI_ISL_406862|2020-01-28) [58, 59] was originally provided by Christian Drosten (Charité, Berlin). It was propagated in Vero B4 cells (ACC 33, DSMZ) and concentrated from cell supernatants using Amicon^®^ Ultra-15 Centrifuge Filters Ultracell^®^ 50KDa (Merck Millipore). The viral preparation used in this study was sequenced using the QiAseq SARS-CoV-2 Primer Panel for Illumina sequencing. The data confirmed the absence of relevant genetic variation that could have arisen from serial passaging in Vero cells (Table S1). The content of replicative virus was determined in Vero B4 cells by plaque assay. All studies involving SARS-CoV-2 infection were conducted within a biosafety level 3 facility at the Hannover Medical School.

Propagation of recombinant HCMV-GFP (strain RV-TB40-BAC_KL7_-SE-eGFP) was performed in MRC5 lung fibroblasts (CCL-171™, ATCC^®^). Cell supernatants were centrifuged for 3 h at 25,000 g and afterwards the virus was purified by 20% sorbitol gradient centrifugation at 10 °C for 75 min at 53,000 g. Viral titers were determined using MRC5, as described elsewhere [60].

### Virus Infection of hiPSC-CMs and hiPSC-MACs

For SARS-CoV-2 infection experiments, 50,000 cm/well in RPMI GlutaMAX medium supplemented with B27, or 20,000 MACs/well in RPMI medium supplemented with 10% FCS, 1% penicillin/streptomycin and 50 ng/ml M-CSF were plated in a 96-well flat bottom plate. Where indicated, cells were treated with the specified concentrations of IFNγ (570206, Biolegend) or IFNα (592706, Biolegend). CMs were infected with SARS-CoV-2 at a multiplicity of infection (MOI) of 0.5 and MACs at MOI 2. After 48 h of incubation, supernatants were collected and kept at − 80 °C until determination of viral titers by plaque assay and cells were fixed with 4% paraformaldehyde (PFA) for 90 min for further immunofluorescence labeling.

For HCMV infection experiments, 0.5 × 10^6^ MACs/well were plated in a 24-well plate with RPMI medium supplemented with 10% FCS, 1% penicillin/streptomycin and 50 ng/ml M-CSF and infected with HCMV-GFP at MOI 3.

### Immunofluorescence Labeling

For detection of SARS-CoV-2 and HCMV infection, CMs and MACs were fixed with 4% PFA for 90 min at RT followed by washing with 1x PBS. Samples were blocked and permeabilized with perm solution [1% BSA, 0.1% Triton, 1.5% Glycine in 1x PBS] for 1 h at RT. Then, a primary antibody cocktail was prepared in labeling buffer [5% BSA, 0.1% Triton in 1x PBS], containing anti-GFP (1:1000, ab6556, Abcam), MMR/CD206 (1:250, MAB2534, R&D), anti-cardiacTroponinT (1:500, ab8295, Abcam), and nucleocapsid (1:500, 40143-T62, SinoBiological). Samples were incubated with 100 µl of the cocktail per well overnight at 4 °C. Afterwards, cells were washed with 1x PBS three times and then incubated for 2 h at RT in the dark with 100 µl of 1:500 diluted secondary antibody Alexa Fluor 594 conjugated donkey anti-rabbit (A21207, Invitrogen) and Alexa Fluor 488 goat anti-mouse (A11017, Invitrogen) for detection of cells infected with SARS-CoV-2, or with Alexa Fluor 488 conjugated donkey anti-rabbit (A21206, Invitrogen) and Alexa Fluor 594 goat anti-mouse (A11032, Invitrogen) for detection of cells infected with HCMV. Cells were washed twice with 1x PBS followed by staining with 1:1000 dilution of Hoechst 3342 (3571, Invitrogen) in 1x PBS for 15 min at RT in the dark, followed by a washing step with 2x PBS. Representative images were captured using a Nikon Ti microscope, and fluorescence intensity analysis was performed using a Cytation 1 (BioTek) device. For the latter, each well was imaged using standard settings, with the device set to auto-adjust for background noise. The background signal (secondary antibody only well) was subtracted from the intensity values before final analysis. These intensity values were plotted and analyzed using GraphPad Prism 8.

### Plaque Assay

To quantify virus titers after SARS-CoV-2 infection, cell-free culture supernatants were collected, subjected to 10-fold serial dilutions and added to Vero B4 cells in a 96-well plate. After 1 h of infection, cells were overlayed with 1% methylcellulose in MEM medium. Upon 72 h incubation at 37 °C, Vero B4 cells were fixed with 10% Formalin for 30 min and viral plaques were stained with crystal violet and counted at the highest viral dilution-containing wells. Analyses were performed in duplicates for each experimental replicate, with plaques counted at the 10^4^ dilution where they were accurately distinguishable. Plaque forming units per milliliter (PFU/ml) of culture supernatants were calculated for each condition.

### Cytokine Measurements in Culture Supernatants

To quantify cytokine concentrations in supernatants derived from infected cultures, a LEGENDplex™ Human Inflammation Panel 1 (740809, Biolegend) kit was used following the manufacturer’s instructions. Cytokine concentrations were measured with a SONY ID7000 spectral flow cytometer and data were analyzed using the LEGENDplex V8.0 software.

## Result

### ACE2 Is Expressed on IFNAR1^comp^ and IFNAR1^def^ CMs, but not on MACs, Which Express NRP1 Regardless of IFNAR1 Competence

**Fig. 1:**
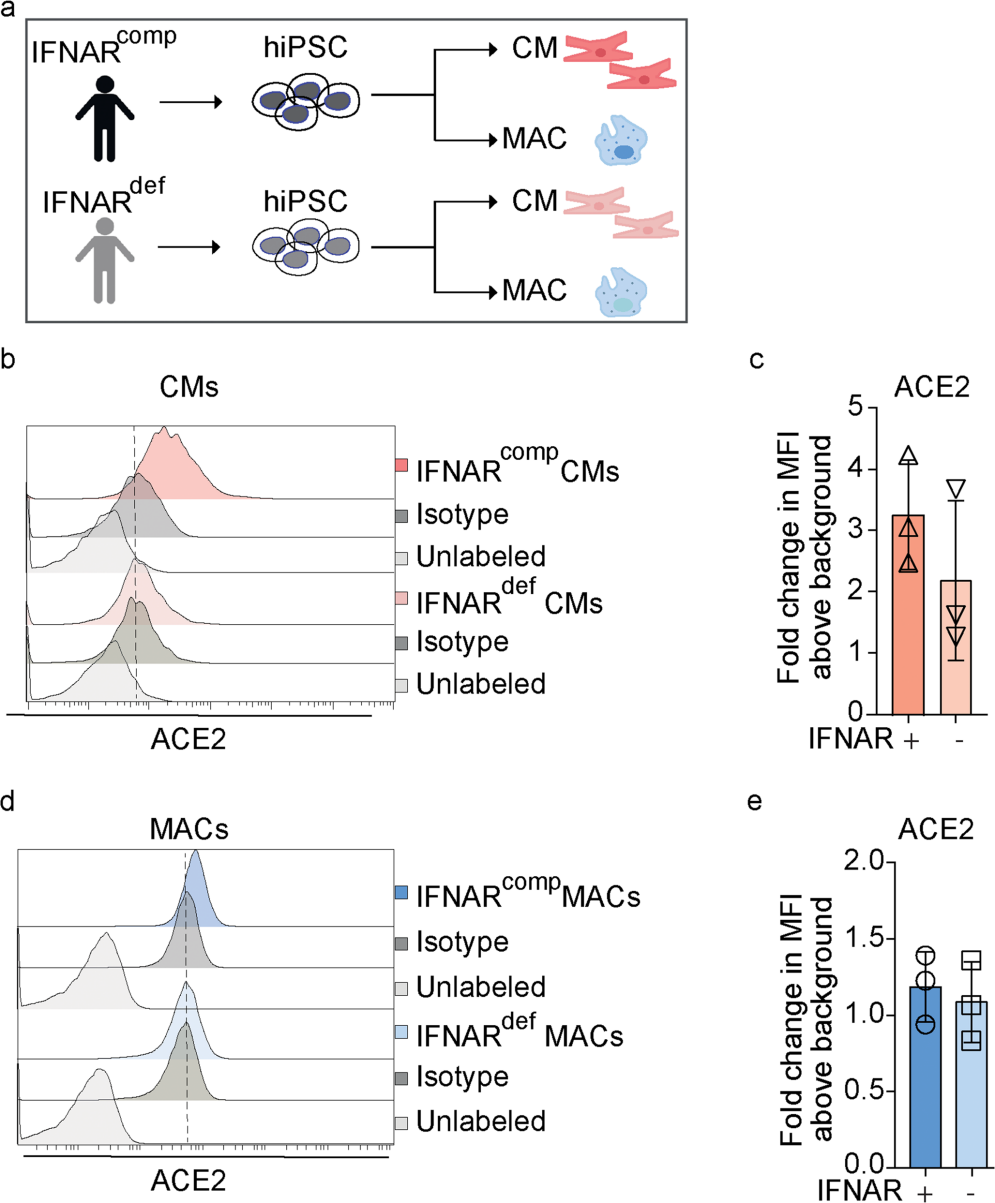
ACE2 is expressed on IFNAR1^comp^ and IFNAR1^def^ CMs, but not on MACs. **a** Schematic depiction of analyzed cells: IFNAR1^comp^ and IFNAR^def^ human iPSC were differentiated to cardiomyocytes [CMs] and macrophages [MACs]. **b** Representative flow cytometry histograms showing surface expression of ACE2 among living propidium iodide negative (PI^neg^) CMs; Light grey: unlabeled cells; Dark grey: cells labeled with isotype control; *n* = 3 independent experiments. **c** Quantification of data in B in fold change of mean fluorescent intensity [MFI] of ACE2 expression above background (isotype control). Error bars indicate mean ± SD; *n* = 3 independent experiments. **d** Histograms show representative surface expression of ACE2 among living propidium iodide negative (PI^neg^) MACs; Light grey: unlabeled cells; Dark grey: cells labeled with isotype control; *n* = 3 independent experiments. **e** Quantification of data in D in fold change of mean fluorescent intensity (MFI) of ACE2 expression above background (isotype control). Data are summarized from three independent experiments (mean ± SD)

To address the impact of IFNAR1 deficiency on SARS-CoV-2 infection in CMs and MACs, we differentiated both cell types from hiPSCs that were derived from either IFNAR1^comp^ or IFNAR1^def^ donors (Fig. 1 A). Successful differentiation was confirmed by transcriptional, phenotypic, and functional characterization of CMs (Fig. S1) and MACs (Fig. S2). No significant differences were observed in cell viability between IFNAR1^comp^ and IFNAR1^def^ cells by propidium iodide staining (Fig. S3 A, B). To evaluate the expression of known SARS-CoV-2 receptors, we performed flow cytometric analysis of ACE2 and qPCR analysis of ACE2 and the neuropilin 1 receptor (NRP1). We found that IFNAR1^comp^ CMs exhibited higher ACE2 expression than IFNAR1^def^ ones, while NRP1 expression was moderate in both cell types (Fig. 1 B, C and Fig. S3 C, D). In contrast, both IFNAR1^comp^ and IFNAR1^def^ MACs showed no detectable ACE2 expression, but higher NRP1 levels than CMs (Fig. 1 D, E and S3 D). Hence, our results indicate higher ACE2 expression in IFNAR1^comp^ than IFNAR1^def^ CMs, while MACs showed abundant NRP1 expression independent of their IFNAR1 status.

### SARS-CoV-2 Does neither Infect IFNAR1^comp^ nor IFNAR1^def^ MACs

**Fig. 2:**
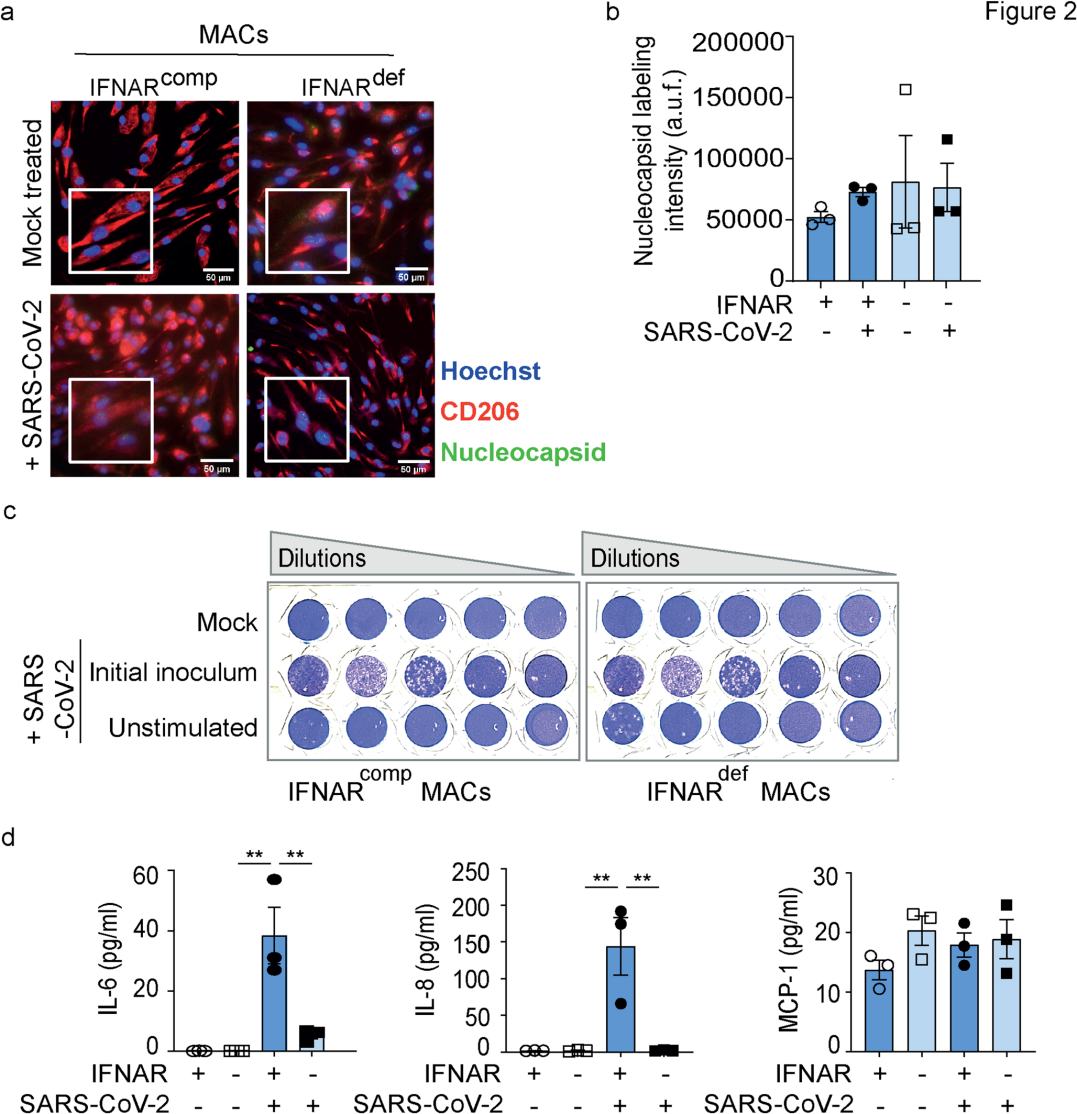
SARS-CoV-2 does not infect neither IFNAR1^comp^ nor IFNAR1^def^ MACs. IFNAR1^comp^ and IFNAR1^def^ MACs were exposed to SARS-CoV-2 at MOI 2 and analyzed after 48 h of incubation. **a** Representative immunofluorescence images of MACs labeled with anti-macrophage mannose receptor (CD206) (red) and anti-SARS-CoV-2 nucleocapsid protein (N) (green). Nuclei were visualized using Hoechst stain (blue). White frames indicate zoomed-in areas. Scale bar, 50 μm. **b** Intensity values of fluorescence signals from the entire well for N quantified from the images in A. Two wells per group, each with 50,000 MACs, were used for analysis; a.u.f = arbitrary unit of fluorescence; Error bars indicate mean ± SEM; *n* = 3 independent experiments. All comparisons between the groups were non-significant; One-way ANOVA, Tukey multiple comparisons test was performed. **c** Representative images from plaque assays where Vero B4 cells were infected with 10-fold serial dilutions of cell-free supernatants derived from uninfected and from unstimulated, SARS-CoV-2 infected MACs. The initial viral inocolum was used as positive control. **d** Cell-free culture supernatants were collected, and the content of IL-6, IL-8 and MCP-1 was determined using a bead-based LEGENDplex™ assay. Error bars indicate mean ± SEM; Supernatants derived from 3 independent experiments. * *p* < 0.05; One-way ANOVA, Tukey multiple comparisons test was performed to check significance between the respective groups. All remaining comparisons between the groups were non-significant

To determine the susceptibility of MACs to SARS-CoV-2 infection, IFNAR1^comp^ and IFNAR1^def^ MACs were exposed to SARS-CoV-2 (MOI 2) for 48 h. Following exposure, cells were labeled for the macrophage mannose receptor (CD206) and for the SARS-CoV-2 nucleocapsid (N) protein, and analyzed by fluorescence microscopy. Across all conditions, we observed abundant CD206 expression, but not detectable N protein (Fig. 2 A, B and S4). Furthermore, cell-free supernatants collected from virus exposed MACs did not contain replication-competent SARS-CoV-2 (Fig. 2 C). Thus, MACs showed no evidence of productive SARS-CoV-2 infection, irrespective of their IFNAR1 expression.

### SARS-CoV-2 Induces IFNAR1-dependent Production of pro-inflammatory Cytokines in MACs

To analyze inflammatory responses of MACs, cells were exposed to SARS-CoV-2 (MOI 2) for 24 h, and cytokines were quantified in cell-free supernatants. IFNAR1^comp^ MACs showed 40 pg/ml and 150 pg/ml of the pro-inflammatory cytokines IL-6 and IL-8, respectively. In contrast, IFNAR1^def^ MACs showed reduced cytokine responses, with 10 pg/ml of IL-6 and absence of IL-8 (Fig. 2 D). MCP-1 levels were similar in IFNAR1^comp^ and IFNAR1^def^ MACs, regardless of exposure to SARS-CoV-2 (Fig. 2 D). Furthermore, stimulation with IFNγ prior to virus exposure reduced IL-6 responses to approximately 10 pg/ml, but did not significantly affect IL-8 production (Fig. S5 B). Therefore, while MACs do not support productive SARS-CoV-2 infection, they are still stimulated by the virus and mount pro-inflammatory cytokine responses, which are higher in IFNAR1^comp^ than in IFNAR1^def^ cells.

### Treatment with Exogenous IFNα Inhibits HCMV Infection of IFNAR1^comp^, but not of IFNAR1^def^ MACs

**Fig. 3:**
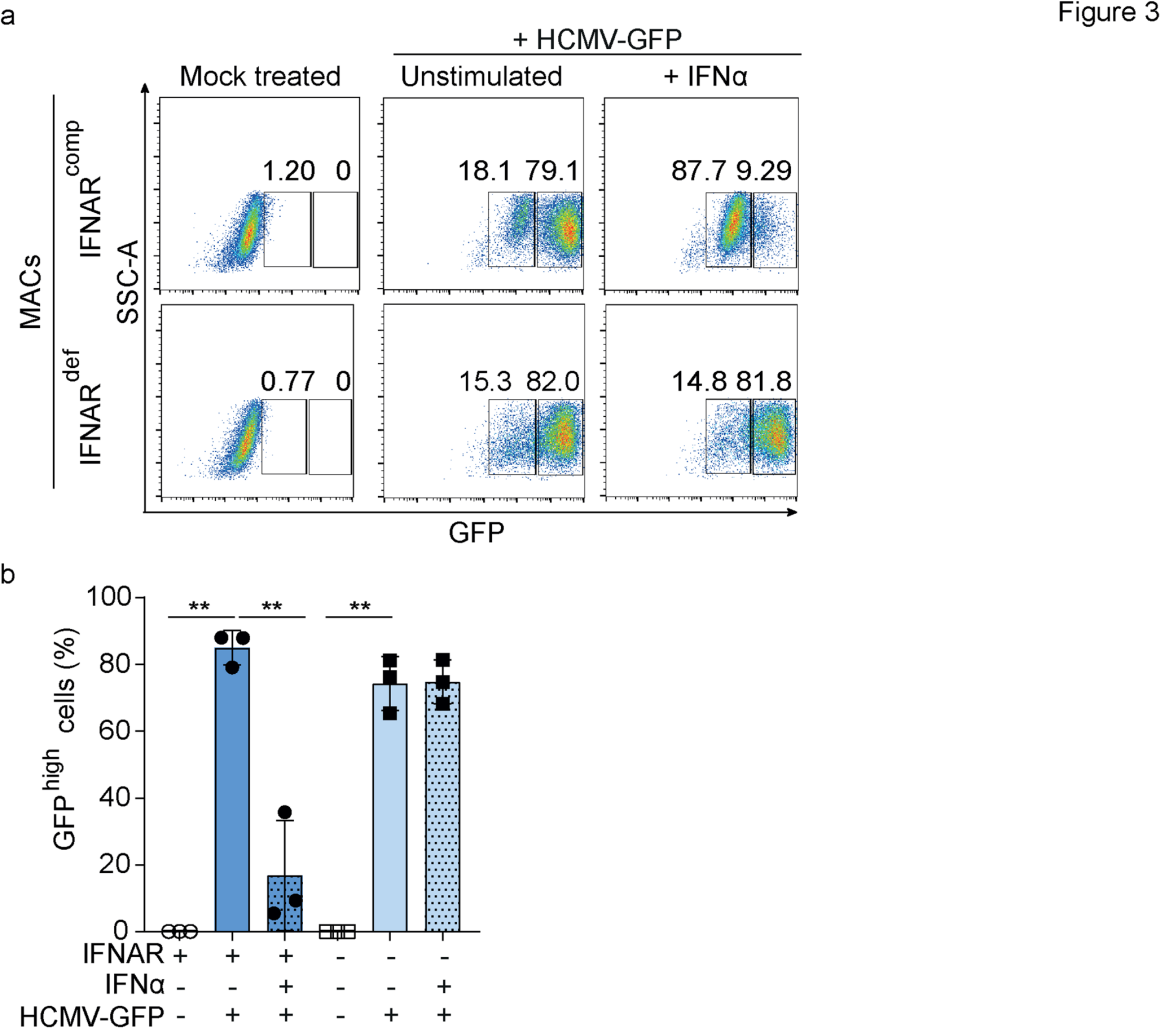
IFNα stimulation inhibits HCMV infection of IFNAR1^comp^, but not of IFNAR1^def^ MACs. IFNAR1^comp^ or IFNAR1^def^ MACs were stimulated with IFNα (25 ng/ml) for 24 h or they were left untreated. Then the cells were infected with HCMV-GFP at MOI 3. **a** Representative flow cytometry dot plots showing the frequency of uninfected (GFP^neg^) and infected (GFP^high^) cells. **b** Frequency of GFP^high^ IFNAR1^comp^ or IFNAR1^def^ MACs upon HCMV-GFP infection with and without IFNα stimulation. Error bars indicate mean ± SD; *n* = 3 independent experiments; ** *p* < 0.01; n.s.= non-significant.; Two-way ANOVA, Tukey multiple comparisons test. All remaining comparisons between the groups were non-significant

To verify the susceptibility of MACs to infection with other viruses, particularly those targeting myeloid cells, we performed infection experiments with human cytomegalovirus (HCMV) expressing the green fluorescent protein under the control of the viral major immediate early promoter (HCMV-GFP) [61]. After exposure of IFNAR1^comp^ and IFNAR1^def^ MACs to HCMV-GFP (MOI 3) and incubation for 24 h, approximately 80% GFP^high^ cells that support viral gene expression were detected, regardless of their IFNAR1 status (Fig. 3 A, B). Upon IFNα stimulation for 24 h and subsequent virus exposure and incubation for another 24 h, IFNAR1^comp^ MACs showed significantly reduced HCMV-GFP infection, with only approximately 20% GFP^high^ cells. Conversely, around 80% of IFNAR1^def^ MACs were GFP^high^, as similarly detected for unstimulated cells (Fig. 3 A, B). These findings indicated that despite their resistance to SARS-COV-2, both IFNAR1^comp^ and IFNAR1^def^ MACs were similarly susceptible to HCMV infection, with IFNα pre-stimulation reducing HCMV infection in IFNAR1^comp^ cells.

### IFNα Stimulation Reduces SARS-CoV-2 Infection in IFNAR1^comp^, but not in IFNAR1^def^ CMs

**Fig. 4:**
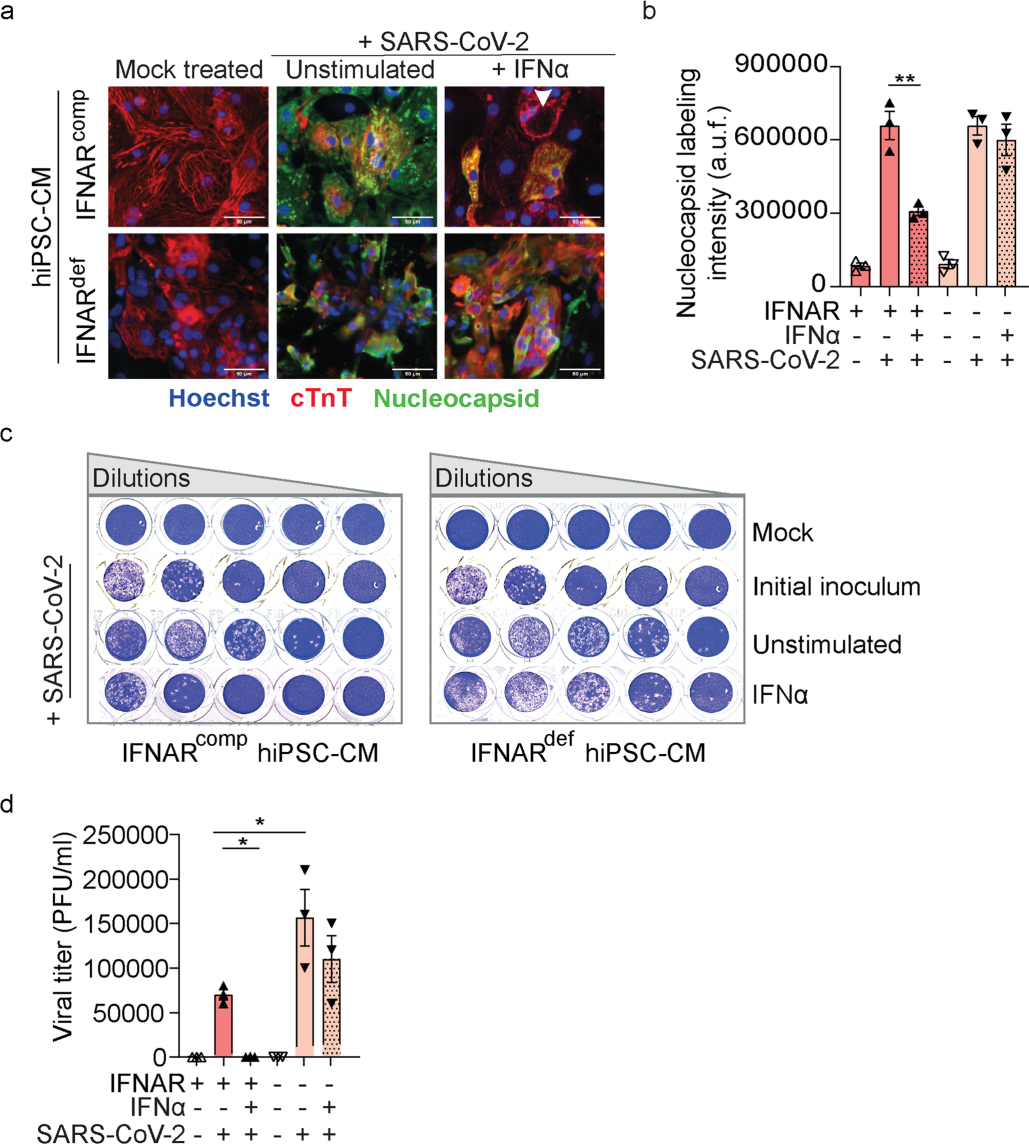
IFNα stimulation inhibits SARS-CoV-2 infection of IFNAR1^comp^, but not of IFNAR1^def^ CMs. IFNAR1^comp^ and IFNAR1^def^ CMs were stimulatedwith IFNα (10 ng/ml) for 24 h or they were left unstimulated. Then, cells were infected with SARS-CoV-2 at MOI 0.5 and analyzed after 48 h of infection. (**a**) Representative immunofluorescence images of CMs after fixing and labeling with anti-cardiac Troponin T (cTnT) (red) and anti-SARS-CoV-2 nucleocapsid protein (N) (green). Nuclei were visualized using Hoechst stain [blue]. White arrowheads indicate less infected IFNAR1^comp^ CMs that were stimulated with IFNα (10 ng/ml, 24 h). Scale bar, 50 μm. **b** Quantification of fluorescence intensity values for SARS-CoV-2 N protein. Two wells per group, each with 50,000 cm, were used for analysis from 3 independent experiments; a.u.f = arbitrary unit of fluorescence. **c** To determine SARS-CoV-2 titers by plaque assay, cell-free supernatants were taken from the cultures, 10-fold serially diluted and transferred on monolayers of Vero B4 cells. Mock supernatant derived from uninfected CMs was used as negative control, and the initial viral inoculum as positive control. Supernatants derived from 3 independent experiments. Representative images are shown. **d** Quantification of plaque forming units [PFU] as observed in C. Error bars indicate mean ± SEM; *n* = 3 independent experiments. PFU/ml = plaque forming units per milliliter. * *p* < 0.05; ** *p* < 0.01; Two-way ANOVA, Tukey multiple-comparisons test. All remaining comparisons between the groups were non-significant

Consistent with the high ACE2 expression, CMs are readily infected by SARS-CoV-2 [62–64]. To assess the effect of IFN-I immunity on SARS-CoV-2 infection, IFNAR1^comp^ and IFNAR1^def^ CMs were treated with IFNα prior to virus exposure, or they were left unstimulated. After 48 h of incubation, CMs were labeled for cardiac troponin T (cTnT) and the SARS-CoV-2 N protein, followed by analysis by fluorescence microscopy. Both IFNAR1^comp^ and IFNAR1^def^ CM exhibited significant cTnT and N labeling (Fig. 4 A, B and S6), indicating a similar level of infection regardless of the IFNAR1 status. Upon IFNα pre-treatment, a marked reduction of N expression was detected in IFNAR1^comp^ CMs, while IFNAR1^def^ cells showed no such effect (Fig. 4 A, B and S6). Therefore, although IFNAR1 deficiency alone did not affect SARS-CoV-2 infection in CMs, exogenous IFNα drastically decreased infection rates in IFNAR1^comp^ but not in IFNAR1^def^ CMs.

### IFNAR1 Deficiency Renders CMs More Susceptible To SARS-CoV-2 Replication

Next, we evaluated SARS-CoV-2 replication by quantifying infective virus particles released to the supernatant. Interestingly, titration of cell-free supernatants from unstimulated IFNAR1^def^ CMs revealed viral loads that were 3-fold higher than those from IFNAR1^comp^ cells (Fig. 4 C, D). Upon IFNα pre-stimulation, IFNAR1^comp^ CMs exhibited 7-fold reduced SARS-CoV-2 titers than unstimulated controls. This effect was not detected in experiments with IFNAR1^def^ CMs (Fig. 4 C, D). Taken together, our data indicated that IFNAR1 deficiency did not affect SARS-CoV-2 infection of hiPSC-CMs, but it affected viral replication. Moreover, IFNα stimulation diminished both infection and replication of SARS-CoV-2, but only in IFNAR1^comp^ and not IFNAR1^def^ CMs.

## Discussion

In this study, we addressed the role of the IFN-I axis in SARS-CoV-2 infection in hiPSC-MACs and CMs. Our findings show that ACE2 expression levels are higher in IFNAR1^comp^ than in IFNAR1^def^ CMs, while being undetectable in MACs. Consistently, MACs did not support productive infection by SARS-CoV-2, yet viral exposure led to a IFNAR1-dependent enhancement of pro-inflammatory cytokine responses, primarily involving IL-6 and IL-8. In CMs, IFNAR1 expression plays a critical role in modulating viral replication, as evidenced by reduced SARS-CoV-2 titers in IFNAR1^comp^ compared to IFNAR1^def^ CMs, despite heightened ACE2 expression in IFNAR1^comp^ cells. Lastly, we showed that stimulation with exogenous IFNα reduced SARS-CoV-2 infection and replication in IFNAR1^comp^, but not in IFNAR1^def^ CMs. Similarly, it inhibited HCMV early viral gene expression in IFNAR1^comp^, but not in IFNAR1^def^ MACs.

ACE2 has been proposed to be an interferon-stimulated gene (ISG) [65, 66]. Thus, it could be hypothesized that endogenous IFN-I accounts for the higher ACE2 levels detected in IFNAR1^comp^ CMs. Interestingly, despite lower ACE2 expression and no significant difference in NRP1 expression, SARS-CoV-2 replication was more pronounced in IFNAR^def^ than in IFNAR^comp^ CMs. These observations raise the possibility that IFNAR1^def^ CMs may support higher viral replication, e.g., by potentially reducing the expression of innate immune sensors that are typically responsible for the control of viral replication [67].This is consistent with recent experimental studies showing that SARS-CoV-2 induces apoptosis and inflammation in human CMs, which can be mitigated by treatment with ACE2 inhibitors such as captopril [68].

While single-cell RNA sequencing (scRNA-seq) studies identified ACE2 transcripts in lung macrophages from COVID-19 patients [69–71], our data revealed no ACE2 expression neither in IFNAR1^comp^ nor IFNAR1^def^ MACs. This aligns with other studies showing that MACs express ACE2 primarily under inflammatory conditions, such as in GM-CSF monocyte-derived MACs, but not in resting monocytes or M-CSF monocyte-derived MACs [38, 72–74]. Despite the shared phenotypic, functional, and transcriptional characteristics of hiPSC-MACs with conventional monocyte-derived MACs, the inbuilt adaptability of myeloid cells to their environment [75–77], which contributes to their phenotypic diversity [78, 79], could explain variation in ACE2 expression among MACs [80, 81]. Besides ACE2-mediated entry, other receptors like NRP1 can facilitate SARS-CoV-2 infection [82]. However, our observations show that neither IFNAR1^comp^ nor IFNAR1^def^ MACs supported SARS-CoV-2 infection, despite of abundant NRP1 expression. To confirm the viral infectability of MACs, we used HCMV, which is known to infect MACs effectively both in vivo and in vitro [83–85]. This is further supported by the high susceptibility of IFNAR1 deficient mice to MCMV, the murine counterpart of HCMV [86, 87].

The role of MACs in modulating inflammatory responses, especially during COVID-19, remains a subject of relevance. Alveolar macrophages have been shown to secrete a broad range of cytokines and chemokines that modulate the lung niche and strongly correlate with severe COVID-19 progression [88, 89]. Yet, the precise dynamics of how MACs contribute to inflammatory responses is not fully understood. Some studies have reported that abortive SARS-CoV-2 infection does not activate inflammatory responses in MACs, and instead, MACs respond to immune signals derived from other infected cells [90, 91]. Others suggested that infected MACs play a direct and significant role in the inflammatory landscape of COVID-19 [80, 92, 93]. Recent in vivo studies have shown that airway-associated interstitial MACs require intact IFNAR1 signaling to control inflammation and viral replication, with conditional deletion of IFNAR1 in CD169+ MACs leading to worsened disease [94]. SARS-CoV-2 targets type II alveolar pneumocytes, the main producers of lung GM-CSF [95]. Correspondingly, alterations in cytokine responses within alveolar macrophages may arise indirectly due to pneumocyte cell death. Moreover, macrophages can acquire viral RNA through phagocytosis of virus-infected cells [96], or by engulfing antibody-coated viral particles via Fc-receptor mediated endocytosis [97].

The use of our in vitro system demonstrated that MACs can initiate a IFNAR1-dependent cytokine response against SARS-CoV-2 infection, independent of the interaction with other infected cell types. We observed an increased production of pro-inflammatory cytokines like IL-6 and IL-8 in IFNAR1^comp^ MACs when compared with IFNAR1^def^ ones. To address whether these differences were specifically associated with SARS-CoV-2 exposure and not due to other factors that might affect cytokine production, we tested the response of both IFNAR1^comp^ and IFNAR1^def^ MACs to HCMV and found no significant differences in the cytokine production of the two cell types (Fig. S7). This suggests that cytokine responses can be modulated by endogenous IFN-I through IFNAR1 interactions, or indirectly via crosstalk with other cytokines, e.g., IFNγ and its receptor (IFNGR) [98]. In this context, delayed IFNAR signalling was shown to have a pathogenic role in a mouse model of SARS-CoV-2 infection [99], leading to severe lung monocytic infiltration and dysregulated cytokine responses.

IFNAR1^def^ CMs showed increased SARS-CoV-2 replication. Moreover, IFNα pre-stimulation did not reduce viral infection, unlike it did in IFNAR1^comp^. These findings highlight the critical role of endogenous and exogenous IFN-I in controlling SARS-CoV-2 infection, particularly in CMs that constitute a natural niche in which SARS-CoV-2 can propagate. It should be noted that other studies reported that pre-treatment with IFNα did not reduce infection in CMs [41]. However, pre-treatment incubation time was only 2 h, and it is known for IFNα that induction of interferon stimulated genes [ISGs] peaks between 6 and 12 h [100]. These data are in line with clinical studies showing severe cases of COVID-19 with symptoms of cardiac damage and dysfunction [42–45, 101]. Notably, SARS-CoV-2 has been shown to impair IFN-I responses by interfering with the formation of the ISGF3 transcriptional complex, which may limit ISG induction even when IFNAR1 signaling is intact [102].Consequently, we hypothesize that some of these cases might be associated with dysregulated IFN-I, in otherwise healthy individuals.

Understanding IFN-I dependent mechanisms might also help to elucidate the pathophysiology of MIS-C, which develops as a COVID-19 related complication in children or young adults, and which is characterized, among other criteria, by cardiovascular dysfunction [103]. The IFNAR^def^ hiPSC technology presented in this study could be used to derive invaluable insights on how to modulate and fine-tune IFN-I responses, particularly between the early and late phase of viral infections. Moreover, it could shed light on the interaction of IFNAR1 with other interferons like IFNγ, or confirm previous findings regarding the benefits of early IFN-I treatment, particularly in patients with known inborn genetic errors that impair IFN-I responses [21].

In summary, our study further emphasizes the necessity of competent IFN-I immunity for the control of SARS-CoV-2 infection, propagation and disease progression. In permissive cells like CMs, deficient IFN-I responses increase susceptibility to infection, providing a niche where SARS-CoV-2 can replicate. In cells that are non-permissive but still sense and respond to SARS-CoV-2 like MACs, IFN-I responses may contribute indirectly to disease progression by dysregulating virus-induced cytokine responses. This can be particularly relevant during the initial stages of infection, as well as at later stages to avoid the hyperinflammation and cytokine storm triggered one or two weeks after initial SARS-CoV-2 exposure [8, 104]. In conclusion, our findings underscore functional implications of the IFN-I system on SARS-CoV-2 exposed cardiomyocytes and macrophages, and provides additional evidence for the association between impaired IFN-I responses and disease severity, with direct clinical relevance.

## Supplementary Information

Below is the link to the electronic supplementary material.

Supplementary Material 1
