# Supplementary material for: Type I IFNs Decrease SARS-CoV-2 Replication in Human Cardiomyocytes and Increase Cytokine Production in Macrophages
Source: J Clin Immunol. 2025 Oct 21;45(1):149. doi: 10.1007/s10875-025-01943-6 (PMC12540622; doi:10.1007/s10875-025-01943-6)
Supplement: Supplementary file 1 — Supplementary Material 1 [file 10875_2025_1943_MOESM1_ESM.pdf]

Type I IFNs decrease SARS-CoV-2 replication in human cardiomyocytes and increase cytokine production in macrophages

Verónica Durán[1,2*], Eirini Nikolouli[3*], Shambhabi Chatterjee[4,5*], Bibiana Costa[1], Andreas Pavlou[1], Annett Ziegler[1], Jennifer Becker[1], Kira Baumann[1], Matthias Bruhn[1], Kathrin Haake[6], Anna Rafiei Hashtchin[6], Ingrid Gensch[3], Andrea Korte[4], Yvonne Lisa Behrens[7], Shen-Ying Zhang[8,9,10], Jean-Laurent Casanova[8,9,10,11,12], Christian Bär[4,5], Nico Lachmann[3,5,13,14*], Thomas Thum[4,5*], Ulrich Kalinke[1,13*#]

Supplementary Information

a

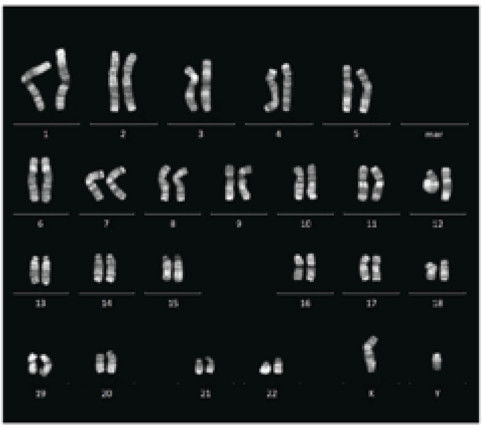

b

Locus	Chromosomal location	Core STR marker	Detected alleles
D3S1358	Chr03	15/17	
TH01	Chr11	Yes	6
D21S11	Chr21	27	
D18S51	Chr18	13/14	
Penta_E	Chr15	13	
D5S818	Chr05	Yes	11/12
D13S317	Chr13	Yes	12
D7S820	Chr07	Yes	9/11
D16S539	Chr16	Yes	10/12
CSF1PO	Chr05	Yes	11
Penta_D	Chr21	10/13	
AMEL	X/Y	Yes	X/Y
vWA	Chr12	Yes	16/18
D8S1179	Chr08	15/16	
TPOX	Chr2	Yes	8/10
FGA	Chr04	20/23	

Fig S1. Genomic analysis of IFNAR^{def} hiPSCs. a) Representative karyotype image of IFNAR1^{def} hiPSCs showing a normal chromosomal complement with no detectable abnormalities. **b)** Short Tandem Repeat (STR) profiling of 15 STR loci and the sex chromosome marker (AMEL). The table presents the locus, chromosomal location, core STR marker status and detected alleles for each marker. This genetic profile is identical between the original IFNAR1^{def} patient-derived fibroblasts and the IFNAR^{def} hiPSC line.

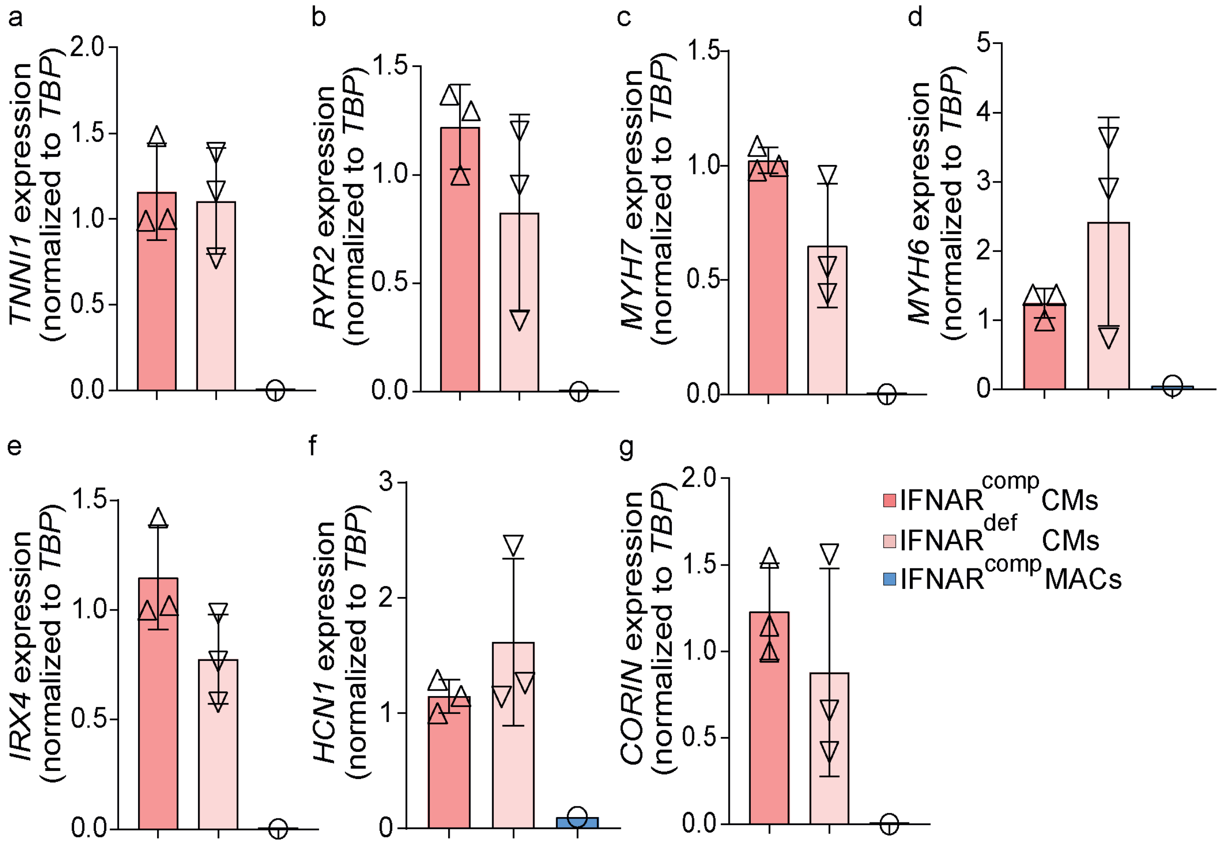

Fig S2. Analysis of cardiac markers in IFNAR1^{comp} and IFNAR1^{def} CMs. Bar graph indicating the mRNA expression of various cardiac-specific genes (**a)** TNNI1, **b)** RYR2, **c)** MYH7, **d)** MYH6, **e)** IRX4, **f)** HCN1, **g)** CORIN) in IFNAR1^{comp} and IFNAR1^{def} CMs compared with IFNAR1^{comp} MACs as a negative control. Error bars indicate mean ± SD.; n= 3 independent differentiation experiments; ** $p < 0.01$; One-way ANOVA, Tukey multiple-comparisons test. All comparisons between the groups were non-significant.

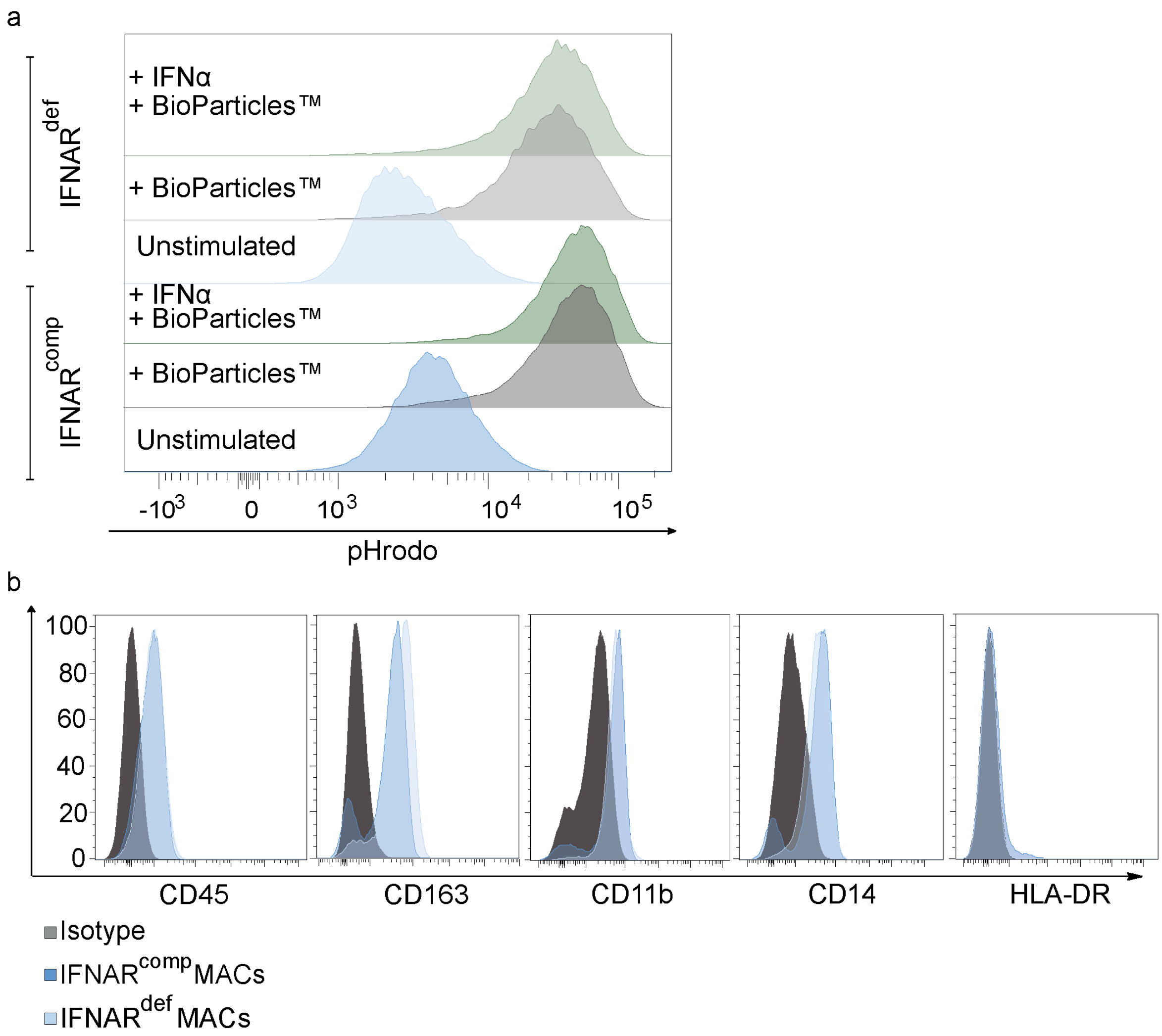

Fig S3. Analysis of macrophage function and expression markers in IFNAR1^{comp} and IFNAR1^{def} MACs. a) pHrodo™ Red *S. aureus* BioParticles™ were added to IFNAR1^{comp} or IFNAR1^{def} MACs, stimulated with or without IFNα (25 ng/ml) and phagocytosis was analyzed. After 3 h of incubation, cells were washed and the mean fluorescence intensity of pHrodo (+part.) was measured by flow cytometry. Representative histograms show a shift in the fluorescence intensity indicating active phagocytosis of the BioParticles™. Unstimulated MACs without pHrodo red (-part.) were used as negative controls. **b)** Cell surface profiling of IFNAR1^{comp} or IFNAR1^{def} MACs was performed by flow cytometry for the macrophage markers CD45, CD163, CD11b, CD14 and HLA-DR. Histograms show representative surface expression of the typical macrophage markers. Note that a small population (<10%) of CD14^{-} immature monocyte/macrophages is present, which represents cells that were not fully matured yet to express CD14. Dark grey: cells labelled with isotype control; n= 3 independent experiments.

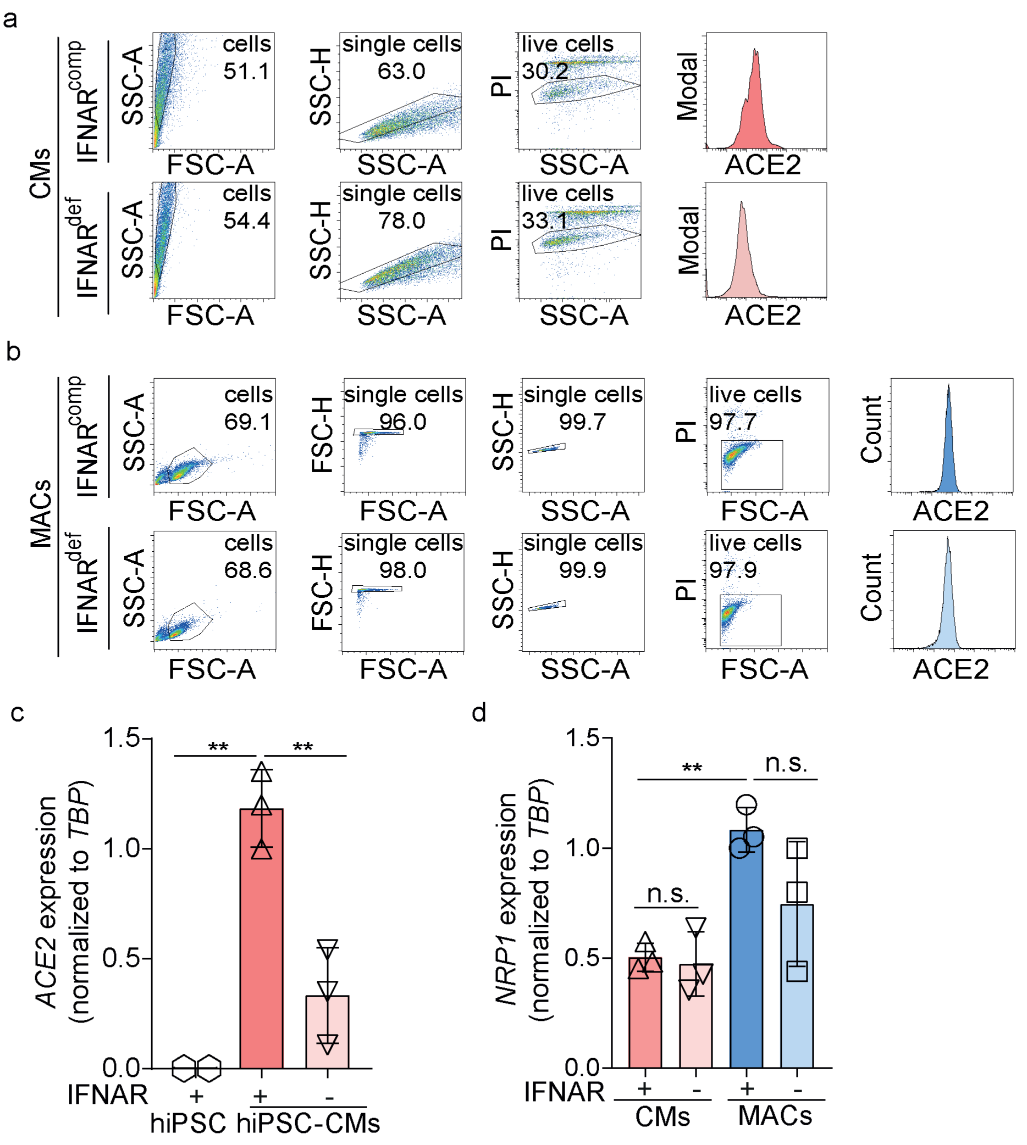

Fig S4. ACE2 and NRP1 expression in IFNAR1^{comp} and IFNAR1^{def} CMs and MACs. a) Gating strategy applied to flow cytometry data to analyze surface ACE2 expression in IFNAR1^{comp} or IFNAR1^{def} CMs. Nucleated cells were gated based on size. Cellular debris and events too small to be cells were excluded from all events. Single cells were gated using SSC-A and SSC-H, thereby excluding doublets. Dead cells stained with propidium iodide (PI) were excluded, and live cells were gated (PI^{neg}). From the PI^{neg} live cell population, the cells were further assessed for ACE2 expression. **b)** Gating strategy applied to flow cytometry data for ACE2 expression analysis in IFNAR1^{comp} and IFNAR1^{def} MACs. Nucleated cells were gated based on size. Cellular debris and events too small to be cells were excluded from all events. Single cells were gated using FSC-A and FSC-H, followed by SSC-A and SSC-H, thereby excluding doublets. Dead cells stained with propidium iodide (PI) were excluded, and live cells were gated (PI^{neg}). From the PI^{neg} live cell population, the cells were further assessed for ACE2 expression. **c)** Bar graph indicating the ACE2 mRNA expression in IFNAR1^{comp} CMs (dark red) or IFNAR1^{def} CMs (light red) compared with undifferentiated hiPSCs. **d)** Bar graph indicating the NRP1 mRNA expression in IFNAR1^{comp} or IFNAR1^{def} CMs compared with MACs (dark and light blue, respectively). Error bars indicate mean ± SD.; n= 3 independent differentiation experiments; ** $p< 0.01$; One-way ANOVA, Tukey multiple-comparisons test.

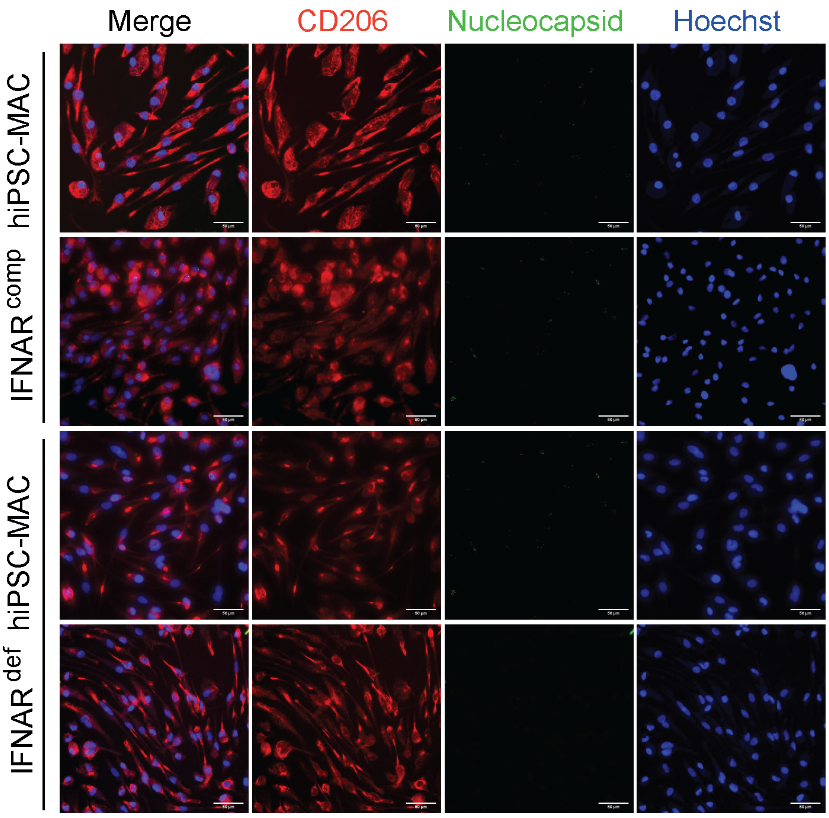

Fig S5. SARS-CoV-2 does not infect neither IFNAR1^{comp} nor IFNAR1^{def} MACs. Representative immunofluorescence images (merged and single) of IFNAR1^{comp} and IFNAR1^{def} hiPSC-MACs labelled with anti-macrophage mannose receptor (CD206) (red) and anti-SARS-CoV-2 Nucleocapsid protein (N) (green). Nuclei were visualized using Hoechst stain (blue). Scale bar, 50 µm.

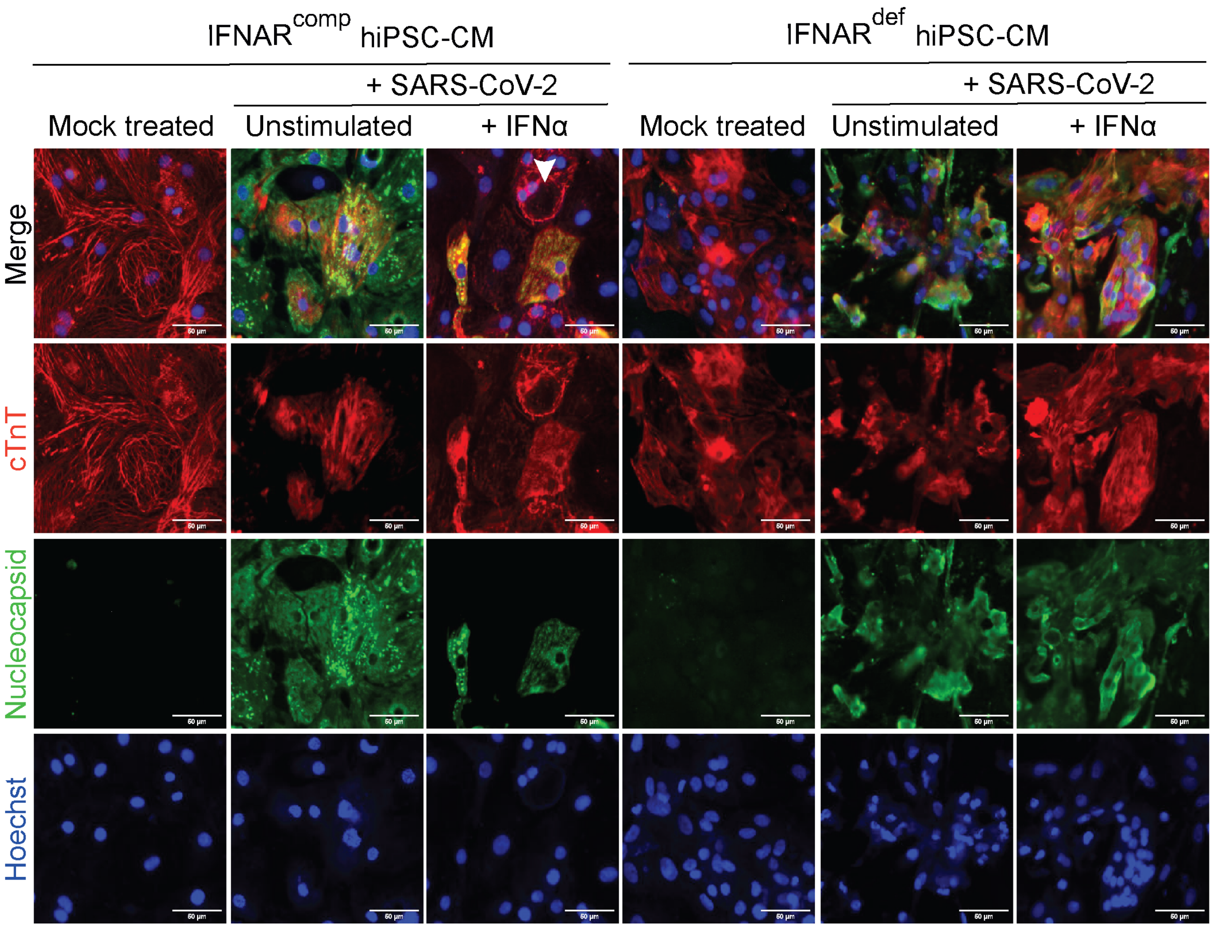

Fig S6. SARS-CoV-2 infects both IFNAR1^{comp} and IFNAR1^{def} CMs. Representative immunofluorescence images (merged and single) of IFNAR1^{comp} and IFNAR1^{def} CMs with anti-cardiac Troponin T (cTnT) (red) and anti-SARS-CoV-2 Nucleocapsid protein (N) (green). Nuclei were visualized using Hoechst stain (blue). Scale bar, 50 µm.

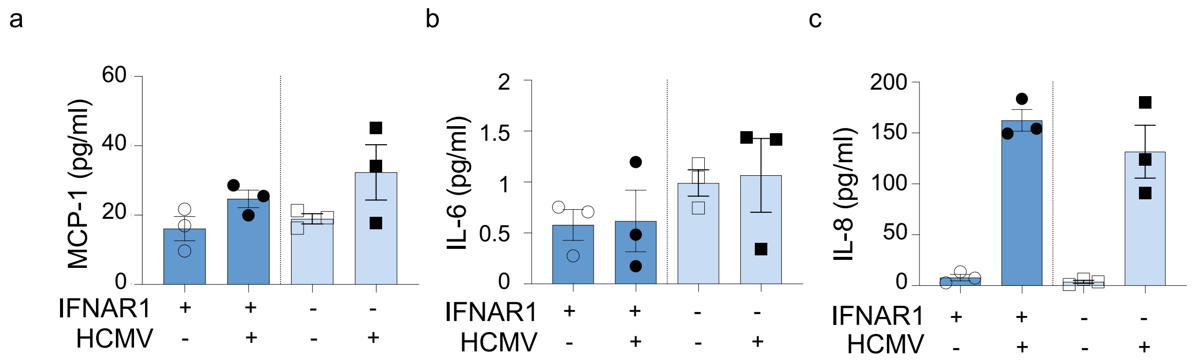

Fig S7. Cytokine production in IFNAR^{comp} and IFNAR^{def} CMs and MACs upon viral stimulation. IFNAR1^{comp} MACs (dark blue) or IFNAR1^{def} MACs (light blue) were infected with HCMV-GFP at MOI 3, cell-free culture supernatants were collected, and the content of **a)** MCP-1, **b)** IL-6 and **c)** IL-8 was determined using a bead-based LEGENDplex™ assay. Error bars indicate mean ± SEM; Supernatants derived from 3 independent experiments. ***p≤0.0002, ****p≤0.0001; One-way ANOVA, Tukey multiple comparisons test was performed to check significance between the respective groups. All remaining comparisons between the groups were non-significant.

Nt position	Ref	Alt	Closest gene	Consequence	Frequency
241	C	U	ORF1ab	noncoding	100%
3037	C	U	ORF1ab	synonymous	99%
16679	C	U	ORF1ab	S5472F	33%
22303	U	G	S	S247R	95%
23403	A	G	S	D614G	100%
23525	C	U	S	H655Y	60%
24965	A	U	S	N1135Y	66%
28906	U	A	N	synonymous	66%

Table S1. Genetic variations in the SARS-CoV-2 preparation used in this study. Genetic characterization of the viral preparation used in this study was performed using the QiAseq SARS-CoV-2 Primer Panel for Illumina Sequencing and the NC-045512.2 as reference genome. The presented single nucleotide polymorphisms (SNPs) are listed by their nucleotide position (Nt position), reference (Ref), and alternate (Alt) alleles, closest gene, consequence and frequency of occurrence (Frequency).
